# Characterization of the *Listeria monocytogenes* PieRS regulon distinguishes the function of the critical secretion chaperone PrsA2 from other regulon members

**DOI:** 10.1128/iai.00357-24

**Published:** 2025-05-27

**Authors:** Xiomarie Alejandro-Navarreto, Laty A. Cahoon, Nancy E. Freitag

**Affiliations:** 1 Department of Microbiology and Immunology, University of Illinois at Chicago 200799, Chicago, Illinois, USA; 2 Department of Biological Sciences, University of Pittsburgh 171653, Pittsburgh, Pennsylvania, USA; 3 Department of Pharmaceutical Sciences, University of Illinois at Chicago 315551, Chicago, Illinois, USA; Universite de Geneve, Genève, Switzerland

**Keywords:** *Listeria monocytogenes*, two-component system, PrsA, bacterial pathogenesis, virulence gene expression, secretion chaperone, intragastric infection

## Abstract

*Listeria monocytogenes* (*Lm*) is a gram-positive pathogen that is widespread throughout the environment and known for its ability to infect mammalian hosts following the ingestion of contaminated food. *Lm* uses a variety of mechanisms to survive challenging conditions experienced both during life in the outside environment and inside of the infected host. We recently described a novel two-component signaling system known as PieRS that regulates the secretion of the chaperone PrsA2, which is essential for bacterial virulence, as well as its related homolog PrsA1 and a variety of gene products of unknown function. Here, we examine the roles of the less characterized PieRS-regulated gene products and contrast their functions with PrsA2 in terms of bacterial survival under stress conditions and virulence in mice. Characterization of targeted in-frame deletion mutants of PieRS regulon members indicates—in contrast to *prsA2* mutants—minimal contributions to stress survival and bacterial virulence. Modest contributions of select regulon members were associated with *Lm* colonization of the gastrointestinal tract. The PieRS regulon thus consists of gene products that contribute to *Lm* physiology in ways that are clearly distinct from PrsA2 and the chaperone’s essential function for both stress survival and bacterial virulence.

## INTRODUCTION

*Listeria monocytogenes* (*Lm*) is a gram-positive non-spore-forming bacterium that is widespread in the environment and able to transition into a pathogen following the ingestion of contaminated food products (1, 2). The ingestion of *Lm* can lead to gastrointestinal symptoms in healthy individuals, while at-risk populations—such as the elderly, immunocompromised patients, and pregnant women—experience more serious invasive diseases leading to meningitis, bacteremia, and stillbirth or abortion (2–4). *Lm* continues to be a significant public health threat, causing sporadic outbreaks worldwide and numerous food product recalls due to *Lm* contamination (5–7).

*Lm* is notable for its ability to survive a wide variety of stress conditions encountered during its life as a saprophyte in soil as well as within the confines of food processing plants and within an infected host. For example, the ability of *Lm* to colonize the gastrointestinal tract requires bacterial resistance to stress conditions that include the acidic pH of the stomach as well as exposure to bile and osmotic stress within the small intestine (3, 8, 9). Mechanisms used by *Lm* to detect environmental insults at the bacterial cell surface and to mount an effective response include the presence of two-component signaling (TCS) systems. TCS systems are typically composed of a bacterial surface histidine kinase that responds to a specific stimulus to initiate a phosphorylation cascade, leading to the activation of a response regulator and subsequent changes in gene expression that contribute to bacterial survival (10, 11). *Lm* has at least 14 TCS systems, of which only a few have been well described (12–14). The systems that have been investigated include those relevant for temperature and antibiotic resistance (LiaSR) (15–17), virulence and ethanol resistance (VirRS) (18), and resistance to osmotic stress (AgrAC) (19). Recently, we described a novel TCS system known as PieRS (PrsA-induced expression system components R and S) that regulates the virulence-associated chaperones PrsA1 and PrsA2 (13). PrsA proteins are secreted chaperones located in the space between the bacterial cell wall and the cell membrane; these chaperones function to fold and stabilize secreted proteins as they are translocated across the cell membrane (20, 21). PrsA2 is critical for the proper folding of a number of *Lm* virulence factors and contributes to cell wall integrity and resistance to a variety of stresses (21). PrsA1 shares 58% amino acid identity and 75% similarity with PrsA2 but appears to play a more limited role in stress resistance and virulence (20–22).

In addition to the critical virulence chaperone PrsA2 and its homolog PrsA1, the TCS system PieRS regulates the expression of several additional gene products of undetermined function (Lmo0442, Lmo0881, Lmo1505, and Lmo1506). Given the common regulation by PieRS, we sought to explore whether these uncharacterized gene products play similar roles in mediating stress resistance and/or bacterial virulence in mice. Here, we describe the initial characterization of these PieRS regulon members through the construction of mutant strains containing in-frame deletions and the comparison of their functions with PrsA2-associated activities.

## RESULTS

### Mutational analyses of PieRS TCS system regulon members

**Fig 1:**
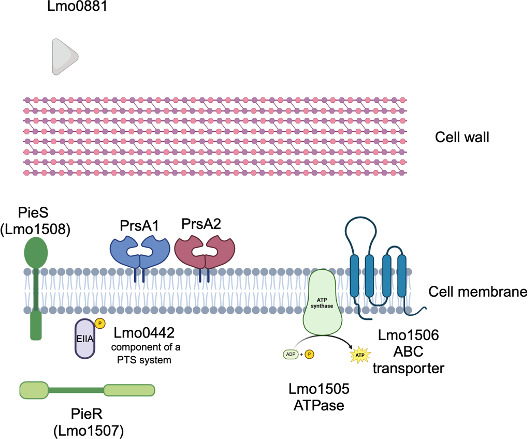
PieRS regulon members. The TCS system PieRS regulates the virulence secretion chaperones PrsA1 and PrsA2. Additional regulon members include Lmo0442, predicted to reside in the cytoplasm; Lmo0881, which appears to have a signal peptide sequence suggesting it is secreted; and Lmo1505 and Lmo1506, predicted to be an ABC transporter and ABC transporter permease, respectively. Image generated using BioRender.com.

Previously, gene members of the PieRS regulon were identified based on DNA microarray and quantitative reverse transcription PCR (qRT-PCR) analyses of wild-type (WT) and overexpression *pieRS* mutant strains (13). In addition to the secretion chaperones PrsA1 and PrsA2, PieRS was found to regulate additional predicted gene products of little-known function: Lmo0442, Lmo0881, Lmo1505, and Lmo1506 (Fig. 1). Lmo0442 has been predicted to function as part of a fructose-specific EIIABC phosphotransferase transport system (PTS) in *Lm* (23, 24). Lmo0881 is a protein of unknown function with an apparent N-terminal signal peptide sequence that suggests the protein is secreted. Lmo1505 and Lmo1506 are predicted to function as an ATP-binding cassette (ABC) transporter and ABC permease, respectively (13).

**Fig 2:**
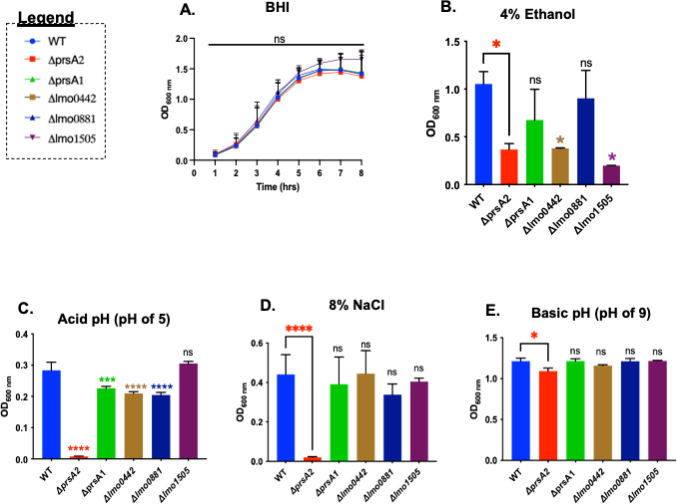
Growth of PieRS regulon mutants in BHI and resistance to stress conditions. (**A**) Wild-type *Lm* and the in-frame deletion mutants were grown in BHI at 37°C without agitation. Then, 1 mL of the normalized overnight culture was added to 24 mL of BHI broth media. Growth was measured by the optical density (OD_600_) every hour for an 8-hour period. For overnight cultures under stress conditions, 2 µL of an overnight culture was inoculated into 2 mL of BHI supplemented with either 4% ethanol (**B**), acid pH (pH 5 with HCl) (**C**), 8% NaCl ([wt/vol] ionic high osmolarity) (**D**), or basic pH (pH 9 with NaOH) (**E**). Cultures were grown at 37°C with agitation (180 rpm), and growth was measured by optical density (OD_600 nm_) the next morning after 18 hours of incubation. Ordinary one-way ANOVA with Dunnett’s multiple comparisons test when compared to wild type with *P* value *≤0.04, ***≤0.0003, and ****≤0.0001. Each regulon member is represented with a color as indicated. The data shown are representative of three independent experiments.

PrsA2, a PieRS regulon member, and, to a lesser degree, PrsA1 have been shown to contribute to *Lm* survival under a variety of different stress conditions, including antibiotic exposure, osmotic stress, and ethanol exposure (13, 20, 25). We, therefore, sought to assess the potential contributions of the other PieRS regulon members to similar stress conditions. In-frame deletion mutants were constructed in wild-type *Lm* using allelic exchange for all genes with the exception of *lmo1506*, for which an in-frame deletion was not successfully obtained. The deletion mutants were first assessed for growth in rich media (brain heart infusion [BHI]). All in-frame deletion mutants exhibited normal growth in BHI media (Fig. 2A). We next tested the ability of the mutants to survive and/or grow in the presence of ethanol, acidic pH, or sodium chloride—conditions that require functional PrsA2—as well as basic pH, for which PrsA2 provides only a modest contribution (Fig. 2). Overnight cultures were normalized based on cell density, diluted into fresh media, and subjected to different stress conditions for 18 hours. The survival/growth was measured by optical density (OD_600_) and compared to the wild-type control and to *ΔprsA2* mutants. Exposure to 4% ethanol reduced the growth of WT *Lm*, with *ΔprsA2* showing a slight increase in sensitivity in comparison, along with *Δlmo0442* and, more notably, *lmo1505* (Fig. 2B). Following exposure to acid pH (pH of 5; Fig. 2C), all strains exhibited a lower cell density when compared with growth at neutral pH, with the *ΔprsA2* strain exhibiting extreme sensitivity. The *Δlmo0442* and *Δlmo0881* mutants exhibited increased sensitivity to acid pH in comparison to WT *Lm*; however, the two mutants remained significantly more resistant than *ΔprsA2*. Previously, the *ΔprsA2* mutant was found to be extremely sensitive to 5% NaCl (13). However, we observed no obvious differences in NaCl sensitivity when testing the other PieRS operon members (X. Alejandro-Navarreto, data not shown). Increasing the NaCl concentration to 8% resulted in reduced growth/survival of the WT strain; however, the PieRS operon mutants exhibited no significant survival/growth differences from WT, even at this increased NaCl concentration (Fig. 2D). With the modest exception of *ΔprsA2*, none of the mutant strains differed from WT *Lm* in their resistance to basic pH (pH of 9; Fig. 2E). Overall, the PieRS regulon members exhibited at most minor differences in stress resistance in comparison to WT *Lm*, while, in contrast, *ΔprsA2* was far more sensitive than WT to most (but not all) stress conditions tested. The *Δlmo1505* mutant was significantly more sensitive to ethanol exposure than WT but exhibited little sensitivity to the other stress conditions tested. PieRS regulon gene member function, therefore, does not appear to extend to all of the same stress responses that require PrsA2 activity for resistance.

### Exploring the contributions of PieRS regulon members to cell wall-related stress resistance

**Fig 3:**
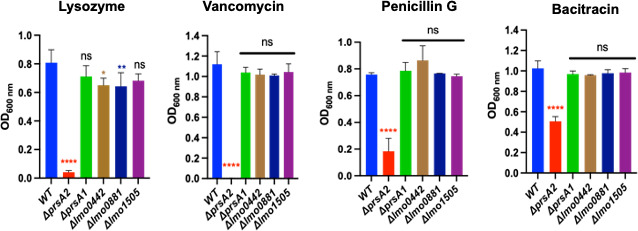
Resistance of PieRS regulon members to cell wall stress conditions. Cultures grown to mid-log phase were inoculated into 2 mL of media containing lysozyme or cell wall targeting antibiotics as indicated and grown at 37°C for 16 hours; bacterial growth was measured using optical density (OD_600 nm_). Lysozyme and antibiotics were used at the following concentrations: lysozyme, 0.5 mg/mL; penicillin G, 0.04 µg/mL; vancomycin, 100 µg/mL; and bacitracin, 100 µg/mL. The data shown are from three independent experiments per condition tested. Ordinary one-way ANOVA with Dunnett’s multiple comparisons test when compared to wild type with *P* value * ≤0.04, ** ≤0.007, and **** ≤0.0001.

Previously, the chaperone PrsA2 was shown to be critical for *Lm* resistance to enzymes and antibiotics that target the cell wall (13, 20). PrsA1 was also found to contribute to cell wall stress resistance but to a lesser degree. We, therefore, explored whether the other PieRS regulon members might similarly have a role in cell wall physiology. We assessed bacterial survival following treatment with lysozyme as well as select antibiotics that target different aspects of cell wall biosynthesis. Antibiotics tested included (i) bacitracin, which affects the recycling of the lipid carrier required for cell wall synthesis (26); (ii) vancomycin, which forms hydrogen bonds with the D-alanyl-D-alanine peptide motif of the peptidoglycan precursor (26); and (iii) penicillin, which targets the penicillin-binding proteins (PBPs) and affects their activity by binding the transpeptidase active domain. If any of the PieRS regulon members contribute to cell wall integrity, the in-frame deletion mutants would be anticipated to exhibit a significant reduction in growth in the presence of cell wall-targeting antibiotics as well as in the presence of lysozyme, which targets peptidoglycan. As anticipated (Fig. 3), the *ΔprsA2* mutant exhibited the most significant sensitivity to the presence of lysozyme, with much more modest reductions exhibited by *Δlmo0442* and *Δlmo0881*, and no significant sensitivity exhibited by either *ΔprsA1* or *Δlmo1505. ΔprsA2* mutants were also observed to exhibit significant sensitivity to penicillin, vancomycin, and bacitracin; in contrast, the remaining mutants exhibited overnight growth patterns under these conditions that appeared similar to WT.

### PieRS regulon members exhibit defects in bacterial entry into tissue culture cells

**Fig 4:**
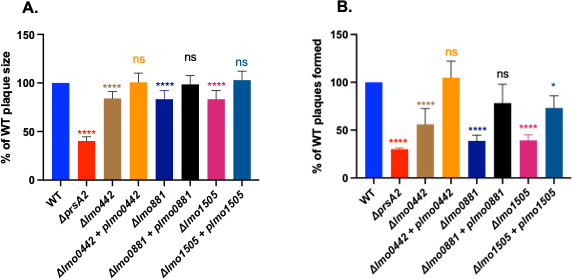
PieRS regulon mutants exhibit defects in plaque formation in L2 fibroblast monolayers. Monolayers of L2 fibroblast cells were infected with 20 µL of overnight normalized cultures of the indicated strains at a multiplicity of infection 10:1. Plaque sizes were measured using a micrometer. (**A**) Plaque sizes relative to wild-type *Lm* and (**B**) the total amount of plaque formation per strain. The data shown are representative of three independent experiments. Ordinary one-way ANOVA with Dunnett’s multiple comparisons test when compared to wild type with *P* value * ≤0.01 and **** ≤0.0001.

*Lm* mutants that lack *prsA2* exhibit dramatic reductions in bacterial virulence in mouse infection models (22, 27). In tissue culture models of infection, the absence of PrsA2 results in significant reductions in bacterial plaque formation and plaque size in L2 fibroblast cells, a phenotype consistent with defects in both bacterial entry into cells and cell-to-cell spread (22). We, therefore, examined the ability of mutant strains lacking other members of the PieRS regulon for plaque formation in L2 fibroblast cells. Cell monolayers were infected with the PieRS regulon in-frame deletion mutants, and plaque formation was assessed after 3 days. Bacteria that successfully invade host cells, replicate intracellularly, and spread to adjacent cells produce zones of cell clearing or plaques. As previously demonstrated, wild-type strains produce numerous plaques in fibroblast cell monolayers, whereas *ΔprsA2* mutants exhibit both a reduction in plaque size (indicating an intracellular replication and/or cell-cell spread defect) as well as reduced numbers of plaques (defects in cell invasion; Fig. 4). The absence of *lmo0442*, *lmo0881*, or *lmo1505* all resulted in reduced numbers of total plaques consistent with cell adhesion and/or entry defects similar to those observed for *ΔprsA2*, as well as slight reductions in plaque size. Complementation of each mutant with a wild-type copy of the gene restored both plaque size and plaque number. These data suggest roles for each regulon member in host cell entry with potentially more minor roles in intracellular growth and/or spread.

### Lmo0881 and Lmo0442 contribute to *Lm* intestinal colonization but are not required for systemic infection

**Fig 5:**
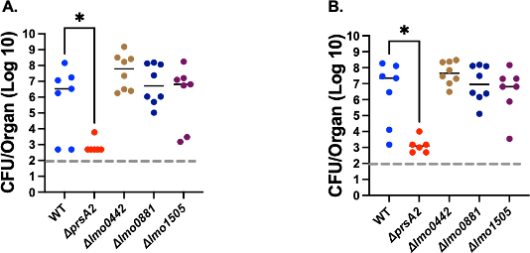
Mouse model of intravenous bacterial infection. Female 6–8 weeks old Swiss Webster mice were infected with 200 µL phosphate-buffered saline (PBS) containing 2 × 10^4^ CFU of bacteria by tail vein injection. Mice were euthanized at 72 hours post-infection, and bacterial burdens were enumerated from isolated livers (**A**) and spleens (**B**). The dashed line (----) in gray represents the limit of detection of CFU, and the black line indicates the median. Only *ΔprsA2* was significantly attenuated, with a *P* value * ≤0.02 using the Kruskal-Wallis (nonparametric) with a suitable post-test, Dunn’s test, in livers (**A**) and spleens (*P* value * ≤0.01) (**B**).

**Fig 6:**
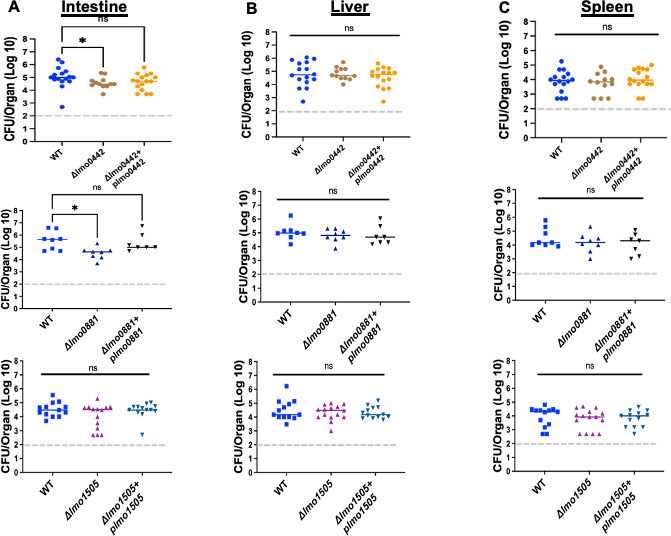
Intragastric infection of Balb/c mice. Six-week-old female Balb/c mice were intragastrically infected with 1 × 10^9^ CFU of the indicated *Lm* strains. At 72 hours post-infection, mice were euthanized, and the bacterial burdens were enumerated in the liver, spleen, and entire intestine (without washing). The dashed line (----) in gray represents the limit of detection of CFU, and the solid color line represents the median of the data. (**A**) Intestine; (**B**) liver; (**C**) spleen. In panel A, *Δlmo0442* and *Δlmo0881* burdens are significantly lower (*P* value *≤0.04 for *Δlmo0442* and *P* value *≤0.015 for *Δlmo0881*) using a Kruskal-Wallis (nonparametric) with a suitable post-test, Dunn’s test.

In keeping with PieRS regulon member comparisons, PrsA2 is known to be required for both oral and intravenous *Lm* infections in mice (13, 27). We, therefore, assessed whether the PieRS regulon members Lmo0881, Lmo0442, and Lmo1505 contribute significant roles to *Lm* mouse infection. Beginning with a systemic infection model, 2 × 10^4^ CFU of WT or the individual deletion mutant strains were injected into the tail vein of female 6–8 week old Swiss Webster mice, and livers and spleens were subsequently harvested and used to enumerate bacterial CFU at 72 hours post-infection. With the exception of the *ΔprsA2* mutant, none of the other deletion mutants exhibited significant defects in the colonization of either mouse liver or spleen (Fig. 5). We next assessed an oral model of mouse infection using female 6-week-old BALB/c mice that were infected intragastrically with 1 × 10^9^ CFU of wild type or individual mutant strains. The *ΔprsA2* mutants were not included in these infections as we have previously shown that the mutant is completely defective for intestinal colonization (13). At 72 hours post-infection, mice were euthanized, and the livers, spleens, and intestines were collected to determine bacterial burdens (Fig. 6). All strains examined yielded comparable burdens in the livers and spleens following oral gavage, whereas mutant strains *Δlmo0442* and *Δlmo0881* exhibited modest but significant decreases in intestinal bacterial burdens in comparison to mice infected with wild-type *Lm. Lm* mutants lacking *lmo0881* exhibited an approximately 10-fold decrease in bacterial CFU by 72 hours post-infection. Each mutant could be successfully complemented with a wild-type copy of the gene (Fig. 6). The failure to detect significant differences in bacteria numbers in distal organs such as the liver and spleen suggests that bacteria that successfully cross the intestinal barrier were fully capable of replicating and spreading within target organs. These data indicate that strains lacking Lmo0442 or Lmo0881 have apparent defects in either intestinal colonization or bacterial survival within the intestine during *Lm* infection, with mutants lacking Lmo0881 exhibiting the most substantial reductions in bacterial numbers.

**Fig 7:**
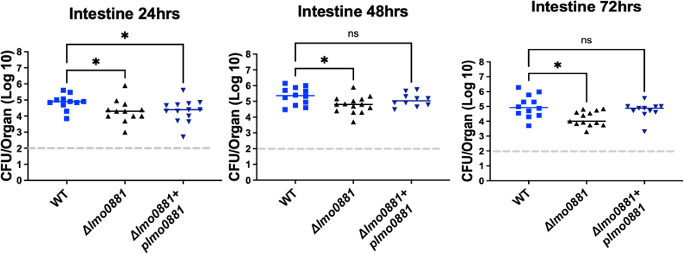
Time course of *Δlmo0881* intragastric infection in Balb/c mice. Balb/c female mice (6-week-old) were infected with 1 × 10^9^ CFU of the indicated bacterial strain using intragastric gavage. At 24, 48, and 72 hours post-infection, intestine samples were collected and processed to enumerate bacterial CFU. Intestines were washed prior to homogenization to distinguish bacteria closely associated with the intestinal epithelium and/or intracellular bacteria from those found within the lumen. The dashed line (----) in gray represents the limit of detection of CFU, and the solid color line represents the median of the data. The Kruskal-Wallis (nonparametric) with a suitable post-test, Dunn’s test with *P* value **≤0.002 and *P* value *≤0.029.

To clarify if the defect observed for the *Δlmo0881* mutant resulted from a defect in initial colonization within the intestine or, alternatively, if persistence within the intestine declined over time, a time course of intragastric infection was conducted using BALB/c mice, which are more sensitive to *Lm* intragastric infection. Mice were intragastrically infected with 1 × 10^9^ CFU of wild type or *Δlmo0881* mutant bacteria. Then intestines were collected at 24, 48, and 72 hours post-infection (Fig. 7). The intestines were washed before being homogenized to distinguish bacteria closely associated with the intestinal epithelium and/or intracellular bacteria from those found within the lumen. Mice infected with *Δlmo0881* exhibited significantly reduced CFU at 48 hours, and the phenotype was even more apparent by 72 hours post-infection (Fig. 7). These results suggest that Lmo0881 makes modest contributions to both the initial bacterial colonization of the intestine as well as bacterial persistence and survival within the intestine.

## DISCUSSION

The TCS system PieRS regulon was recently identified based on its role in regulating the expression of the secretion chaperones PrsA1 and PrsA2, both of which contribute to *Lm* virulence and stress resistance (13). In addition to *prsA1* and *prsA2*, PieRS was also found to regulate the expression of four additional genes of unknown function: *lmo0442*, *lmo0881*, *lmo1505*, and *lmo1506*. Here, we used deletion analyses of three of the four genes to determine if these lesser-characterized PieRS regulon members make similar contributions to *Lm* stress resistance and virulence. Modest contributions for resistance under severe acid stress conditions were observed for Δ*lmo0442*, Δ*lmo0881*, and Δ*lmo1505*, together with a significant role for Δ*lmo1505* under conditions of ethanol stress. All of the deletion mutants exhibited host cell adhesion and/or invasion defects based on the reduced numbers of plaques formed in fibroblast tissue culture cell infection models, and *lmo0442* and *lmo0881* gene products were found to make modest contributions to intestinal colonization/survival in mouse models of intragastric infection. In contrast to what has been observed for *prsA2*, Lmo0442 and Lmo0881 mutants had no discernible defects in virulence associated with systemic infection. Our results indicate that the members of the PieRS regulon differ from PrsA2 with respect to their roles in stress resistance and virulence, with contributions that are independent of those of PrsA2 and, in most cases, of lesser magnitude.

We recognize that a limited number of stress conditions were tested to assess gene function, and the full contributions of the PieRS regulon to *Lm* physiology remain to be determined. The environmental signal(s) that lead to PieRS activation have not yet been identified, and understanding what triggers PieRS activation would undoubtedly help clarify regulated gene product function. It is also possible that the expression of *lmo0442*, *lmo0881*, *lmo1505*, and *lmo1506* may be regulated by additional factors, similar to the regulation of *prsA2* by both PieRS and PrfA, the central regulator of *Lm* virulence. *prsA2* appears unique among regulon members with respect to PrfA regulation, yet while PrsA2 function is absolutely required for virulence, Zemansky et al. (28) have shown that PrfA regulation of *prsA2* is not essential, indicating the importance of alternative or redundant forms of *prsA2* regulation.

*Lm* is known for its stress resistance, and this organism has been demonstrated to possess multiple pathways and systems for dealing with a wide range of stress conditions (29–31). For example, additional TCS systems have been shown to contribute to *Lm* stress resistance, including LisRK which contributes to resistance to cell wall targeting antibiotics; LiaSR (regulated by LisRK) associated with resistance to agents that perturb the cell envelope; and CesKR, associated with resistance to bile, detergents, and ethanol (14). Redundancy appears to exist such that the loss of one regulatory pathway can often be compensated for by another system (13, 17). Interestingly, CesKR appears to regulate genes that are located adjacent to the PieRS regulon member Lmo0442 (13, 32). It is thus possible that, as mentioned above, some members of the PieRS regulon have overlapping functions with other TCS system regulon members.

In the process of constructing the in-frame deletion mutants, we were unsuccessful in generating the *lmo1506* in-frame deletion (*Δlmo1506*). The reason for this failure is unclear. Lmo1506 is predicted to be an ABC-transporter permease potentially associated with the Lmo1505 ATP-binding protein. We were successful in generating plasmid insertion mutations within *lmo1506*, indicating that the gene product is not essential for bacterial growth (X. Alejandro-Navarreto, data not shown). However, the process of allelic exchange using recombination is such that any mutations that confer a fitness defect are difficult to isolate, given that bacteria containing the wild-type copy of the gene tend to overgrow the culture. In future studies, it may be possible to generate the deletion mutation by replacing the deleted copy of the gene with a selectable antibiotic resistance cassette; this was not pursued in the current work.

*In vivo* mouse data suggest that Lmo0442 and Lmo0881 both have modest roles in colonization and/or survival within the intestine. Lmo0442 has previously been suggested to be an enzyme IIA (EIIA) component of a PTS carbohydrate uptake system associated with fructose uptake (13, 23, 33). However, the *Δlmo0442* did not exhibit any defects when grown on fructose as a sole carbon source (X. Alejandro-Navarreto, data not shown). Analysis of the predicted gene products adjacent to *lmo0442* reveals that *lmo0441* appears to be transcribed in the opposite direction of *lmo0442* and is regulated by the TCS CesKR, associated (as mentioned above) with resistance to bile, detergents, and ethanol (32, 34, 35). Lmo0441 encodes a PBP and has been shown to be relevant under conditions of ethanol and ß-lactam antibiotic stress (13, 32). CesRK also appears to regulate *lmo0443*. The Lmo0443 gene product is a member of the LytR-CpsA-Psr (LCP) family of proteins and is induced by the TCS CesRK in response to cell wall damage (32, 36). Lmo0446 has been described as a cholylglycine hydrolase family protein that catalyzes the hydrolysis of glycine as well as conjugated bile acids (37). Bile is abundantly produced in the gallbladder and secreted into the intestine to aid in the breakdown and absorption of fat and also acts as a host defense mechanism against pathogens colonizing the GI tract (3, 8). Lmo0447 and Lmo0448 are members of the glutamate decarboxylase family of proteins that have roles during acid stress resistance, especially when *Lm* encounters acidic pH during the colonization in the host (3, 38). It appears, therefore, that *lmo0442* is located near genes encoding proteins relevant to bacterial survival under stress conditions such as bile resistance and acid pH, the very conditions that *Lm* encounters during the colonization of the intestine (3, 8). The actual role of Lmo0442 within the intestine remains to be determined.

Lmo0881 is predicted to be a secreted protein, and a time course of mouse intragastric infection indicates that Lmo0881 contributes to intestinal colonization within the first 24 hours of infection. The use of the Alpha Fold structural program predicts that Lmo0881 shares structural homology with an immunoglobulin-like domain predicted to be important for protein-protein interactions (X. Alejandro-Navarreto, data not shown). It is possible that Lmo0881 works in concert with a protein-binding partner, possibly of bacterial or host origin, which might contribute to *Lm* colonization of the intestine. Future experiments to clarify the role of Lmo0881 in intestinal colonization/survival may provide additional mechanisms based on the identification of potential Lmo0881 protein-binding partners. Overall, the experiments presented here represent the first efforts toward gaining a better understanding of the roles of PieRS regulon members beyond the known roles of the secretion chaperones PrsA1 and PrsA2; future studies are needed to further unravel the precise contributions of these lesser-known regulon members.

## MATERIALS AND METHODS

### Bacterial strains, plasmids, and media

All the bacterial strains used in this study are described in Table 1. *Lm* 10403S is the wild-type strain (NF-L100), and *ΔprsA2* is *Lm* 10403S containing a complete deletion of *prsA2* with an erythromycin cassette (erm) in place of the *prsA2* gene (NF-L1651) (22). *Escherichia coli* One Shot TOP10 (Invitrogen), high-efficiency 5-alpha competent *E. coli* (NEB), and S17 (kindly provided by N. Cianciotto, Northwestern University) were used as host strains for recombinant plasmids. *Lm* and *E. coli* strains were grown in BHI or Luria broth (LB) media. For genetic complementation studies, the plasmid pIMK2 (39) was used to express the wild-type copy of the gene for the strains *∆lmo0442*, *∆lmo0881*, and *∆lmo1505*.

**TABLE 1:** Strains and plasmids

Strain	Genotype	Designation	References
Top 10	*E. coli* strain with recombinant pKSV7 plasmid		(40)
Top 10	*E. coli* strain with recombinant pIMK2 plasmid		(39)
S17	*E. coli* conjugation recombinant pIMK2 plasmid		(39)
NF-L100	*Lm* 10403S parent strain	WT	(41)
NF-L1651	*Lm* 10403S *ΔprsA2::erm*	*ΔprsA2*	(22)
NF-L18	*Lm* 10403S *ΔprsA1*	*ΔprsA1*	(20)
NF-4909	*Lm* 10403S in-frame deletion of *lmo0442*	*Δlmo0442*	This work
NF-4911	*Lm* 10403S in-frame deletion of *lmo0881*	*Δlmo0881*	This work
NF-4913	*Lm* 10403S in-frame deletion of *lmo1505*	*Δlmo1505*	This work
NF-4942	*Lm* 10403S complemented strain using pIMK2 with wild-type copy *lmo0442*	*Δlmo0442 + plmo0442*	This work
NF-4944	*Lm* 10403S complemented strain using pIMK2 with wild-type copy *lmo0881*	*Δlmo0881 + plmo0881*	This work
NF-4946	*Lm* 10403S complemented strain using pIMK2 with wild-type copy *lmo1505*	*Δlmo1505 + plmo1505*	This work

### Construction of in-frame deletion mutants and complemented strains

The in-frame deletions of the regulon members were constructed using splicing by overlap extension PCR (42). Two DNA fragments were generated by PCR using primers A/B and C/D (described in Table 1) for each regulon member and amplified using genomic DNA isolated from *Lm* 10403S. The two fragments for each gene were gel purified (Qiagen) and PCR amplified using primer pair A/D (Table 1) to generate the following products*: ∆lmo0442*, 1,536 bp; *∆lmo0881*, 1,537 bp; and *∆lmo1505*, 1,487 bp. Each deletion construct was cloned into the shuttle vector pKSV7 (43) and introduced into WT *Lm* to generate mutants using allelic exchange as previously described (40). Complementation vectors for deletion mutants were generated by PCR amplification of the WT genes from genomic *Lm* 10403S DNA using primers described (Table 2). The PCR-amplified genes were individually cloned into the integration plasmid pIMK2 (39), providing for constitutive gene expression. Plasmid constructs were transformed into S17 cells and then transferred into the appropriate *Lm* mutant strains via conjugation, and allelic exchange was used to generate *Lm* deletion mutations (39). All mutants were confirmed using DNA sequencing.

**TABLE 2:** Primers used for cloning

Gene	Primer name	Primer sequences (5′−3′)	References
*lmo0442*	SOE442SACF1A	AGTA**GAGCTC**CCCCTTCACCAAGCTTCGAA	This work
*lmo0442*	SOE442ClaB	GATTTCTAGAGGGCTCGCAA**ATCGAT**CCAACATCATCCTTTCGTTAC	This work
*lmo0442*	SOE442ClaC	GTAACGAAAGGATGATGTTGG**ATCGAT**TTGCGAGCCCTCTAGAAATC	This work
*lmo0442*	SOE442XMAR1D	AGTA**CCCGGG**CTCCTTCTGCATCTTTACGG	This work
*lmo0881*	SOE881SACF1A	AGTA**GAGCTC**CCGGCAAATAGCCGTTACTA	This work
*lmo0881*	SOE881ClaB	CATTTTATTTAGAGTGCTTCC**ATCGAT**CATTCTCACCTCTTCTTGTT	This work
*lmo0881*	SOE881ClaC	AACAAGAAGAGGTGAGAATG**ATCGAT**GGAAGCACTCTAAATAAAATG	This work
*lmo0881*	SOE881XMAR1D	AGTA**CCCGGG**TACGGATACGCTAGGTTCAG	This work
*lmo1505*	SOE1505SACF1A	AGTA**GAGCTC**CACGGTAGACTCAGCTGTTT	This work
*lmo1505*	SOE1505ClaB	AAAGAACGGATGGCTAAATG**ATCGAT**CGTCACCTACTCCTGTTTAG	This work
*lmo1505*	SOE1505ClaC	CTAAACAGGAGTAGGTGACG**ATCGAT**CATTTAGCCATCCGTTCTTT	This work
*lmo1505*	SOE1505XMAR1D	AGTA**CCCGGG**TCCAATACCGATATCCCAG	This work
*lmo0442*	*lmo0442*compSacIF1	AGTA**GAGCTC**GAAAACTTTAGATTTGCCCG	This work
*lmo0442*	*lmo0442*compXmaIR1	AGTA**CCCGGG**AGCCCCCTAAAATGATGATT	This work
*lmo0881*	*lmo0881*compSacIF1	AGTA**GAGCTC**AAACAGCTAAATTGGTAGCG	This work
*lmo0881*	*lmo0881*compXmaIR1	AGTA**CCCGGG**ATTTTATTTAGAGTGCTTCC	This work
*lmo1505*	*lmo150*5compSacIF1	AGTA**GAGCTC**GAATTGGGATCGGAATTGCC	This work
*lmo1505*	*lmo1505*compXmaIR1	AGTA**CCCGGG**AATTATAAAGAACGGATGGC	This work

### Bacterial growth curves analysis

For overnight cultures, 2 mL aliquots of BHI broth were inoculated with the appropriate strain from glycerol stocks, and cultures were grown overnight without agitation at 37°C. For growth curve assays, 1 mL of overnight culture was inoculated into 24 mL (1:25) of BHI. Cultures were grown at 37°C with agitation (180 rpm), and growth was measured by optical density (OD_600 nm_) every hour for a total of 8 hours.

### Bacterial survival under stress conditions

Overnight cultures were inoculated with the appropriate strain from glycerol stocks into 2 mL of BHI broth culture and grown without agitation at 37°C. The next day, 2 µL of the overnight culture was inoculated into 2 mL of BHI supplemented with either 4% ethanol, 8% NaCl ([wt/vol]; ionic high osmolarity), acid pH (pH five with HCl), or basic pH (pH nine with NaOH). Cultures were incubated at 37°C with agitation (180 rpm), and growth was measured by optical density (OD_600 nm_) the next morning after 18 hours of incubation.

### Bacterial resistance to agents that target the bacterial cell wall

Bacterial resistance to agents that target the integrity of the bacterial cell wall was determined by inoculation of 2 µL mid-log-phase (OD_600_ of ∼0.8) cultures into 2 mL BHI broth in 4 mL polypropylene test tubes containing dilutions of bacitracin, vancomycin, penicillin G, or lysozyme. The following concentrations were used: lysozyme, 0.5 mg/mL; penicillin G, 0.04 µg/mL; vancomycin, 100 µg/mL; and bacitracin, 100 µg/mL. Cultures were grown at 37°C for 16 hours, and sensitivity to the presence of lysozyme or antibiotic was assessed based on optical density (OD_600 nm_).

### Plaque assays

Monolayers of L2 fibroblast cells were grown to confluency in six-well dishes. Bacteria cultures were grown without shaking at 37°C in BHI broth. L2 monolayers were infected with 20 µL of phosphate-buffered saline (PBS)-washed overnight normalized cultures at a multiplicity of infection of 10:1 (bacteria:cells). After 60 minutes of incubation of the bacteria with the L2 monolayers, the media were removed by aspiration, and the monolayers were washed twice with 37°C Dulbecco’s phosphate-buffered saline (DPBS). Pre-warmed 2× Dulbecco’s Modified Eagle Medium (DMEM), mixed with 1.4% agarose in a 1:1 ratio and with a final concentration of 10 µg/mL of gentamycin, was then added to each well in a final volume of 2 mL. Three days after infection, neutral red was mixed with pre-warmed 2× DMEM mixed with 1.4% agarose in a 1:1 ratio added to each well in a final volume of 2 mL, and plaques were visualized and measured with a micrometer.

### Mouse model of intravenous and intragastric infections

Animal procedures were IACUC approved and performed in the Biological Resources Laboratory at the University of Illinois at Chicago. Overnight cultures of the appropriate strains were diluted 1:20 in BHI broth media and grown to an OD_600 nm_ of 0.75 and normalized to 1. Then, 1 mL of culture (corresponding to ~6 × 10^8^ CFU/mL) was washed twice in sterile DPBS pH 7 and re-suspended in DPBS pH 7 to a final concentration of 1 × 10^5^ CFU/mL. Female 6–8 weeks old Swiss Webster mice (Charles River Laboratories) were inoculated with 200 µL containing 2 × 10^4^ CFU of bacteria by tail vein injection. For oral infections, 6-week-old BALB/c mice (Charles River Laboratories) were infected with 200 µL containing 1 × 10^9^ CFU of bacteria by gastric gavage. For intragastric infections, the intestines, livers, and spleens of infected animals were isolated for bacterial enumeration. In the case of the infected intestine, LB media with 200 µg/mL of streptomycin were used to select for *Lm* and to determine the total CFU. For intravenous injection, the liver and spleen were isolated for bacterial enumeration. Collected organs were placed in 5 mL of sterile H_2_O and homogenized; bacteria burdens were assessed by plating 10-fold serial dilutions on LB media to determine the total CFU.

### Time course of intragastric mouse infection model

Overnight cultures of the appropriate strains were diluted 1:20 in BHI broth media and grown to an OD_600 nm_ 0.75 and normalized to 1. Then, 1 mL of culture (corresponding to 6 × 10^8^ CFU/mL) was washed twice in sterile DPBS pH 7, diluted, and re-suspended in DPBS pH 7 to a final concentration of 1 × 10^5^ CFU/mL. For oral infections, 6-week-old female BALB/c mice (Charles River Laboratories) were infected with 200 µL containing 1 × 10^9^ CFU of bacteria by gastric gavage. At 24, 48, and 72 hours, intestines were collected to assess bacterial CFUs. The intestines were washed before being homogenized with 5 mL of sterilized DPBS. Bacteria burdens in organs were determined by making 10-fold serial dilutions that were plated in LB media with 200 µg/mL of streptomycin to select for *Lm* and to determine the total CFU.

### Statistical analysis

Using the GraphPad software, an ordinary one-way ANOVA with Dunnett’s multiple comparisons test was used to compare all the mutant means to wild-type control with a *P* value ≤ 0.05 considered significant. This approach was used to analyze data for the survival graphs of PieRS regulon members, resistance to cell wall targeting agents assay, and L2 fibroblast plaque data. The *in vivo* mouse data were log transformed, then Kruskal-Wallis (nonparametric) was used with Dunn’s test, with a *P* value ≤ 0.05 considered significant.
